# Secularity, abortion, assisted dying and the future of conscientious objection: modelling the relationship between attitudes

**DOI:** 10.1186/s12910-019-0408-4

**Published:** 2019-09-18

**Authors:** Morten Magelssen, Nhat Quang Le, Magne Supphellen

**Affiliations:** 1 Centre for Medical Ethics, Institute of Health and Society, University of Oslo, Pb. 1130 Blindern, N-0318 Oslo, Norway; 2 SNF Centre for Applied Research at NHH, Bergen, Norway; 3 Department of Strategy and Management, Norwegian School of Economics, Bergen, Norway

**Keywords:** Abortion, Assisted dying, Conscientious objection, Euthanasia, Public attitudes

## Abstract

**Background:**

Controversies arise over abortion, assisted dying and conscientious objection (CO) in healthcare. The purpose of the study was to examine the relationship between attitudes towards these bioethical dilemmas, and secularity and religiosity.

**Method:**

Data were drawn from a 2017 web-based survey of a representative sample of 1615 Norwegian adults. Latent moderated structural equations modelling was used to develop a model of the relationship between attitudes.

**Results:**

The resulting model indicates that support for abortion rights is associated with pro-secular attitudes and is a main “driver” for support for assisted dying and opposition to conscientious objection.

**Conclusions:**

This finding should be regarded as a hypothesis which ought to be tested in other populations. If the relationship is robust and reproduced elsewhere, there are important consequences for CO advocates who would then have an interest in disentangling the debate about CO from abortion; and for health systems who ought to consider carefully how a sound policy on CO can safeguard both patient trust in the services and the moral integrity of professionals. It is suggested that if religiosity wanes and pro-secular and pro-abortion attitudes become more widespread, support for CO might decline, putting into question whether present policies of toleration of conscientious refusals will remain acceptable to the majority.

## Background

Conscientious objection (CO), where a healthcare professional refuses to participate in healthcare service provision for moral or religious reasons, is likely to become an increasingly prominent dilemma in jurisdictions and healthcare systems in the Western world [1]. With increasing medical possibilities come more controversial procedures and moral grey zones, including but not limited to assisted reproductive techniques, genetic engineering, therapy based on embryo-destructive research, and prenatal screening. Such dilemmas are likely to give rise to moral qualms among subsets of practitioners, and, for some, to refusals to participate in specific procedures: conscientious objection. Such novel cases of CO would then supplement the traditional cases of refusals to participate in abortions, limitation of life-prolonging treatment, or assisted dying (euthanasia and physician-assisted suicide) [1].

Trust is a prime resource necessary for health provider systems to thrive [2–5]. Policies for tolerance for conscientious objection might protect public trust, yet might also damage trust, depending on public perception. The CO of subsets of employees might therefore lead to difficult balancing acts for health systems: On the one hand, health systems must protect the professional autonomy and moral integrity of employees, and must respect any statutory rights for CO to be tolerated in specific situations and on specific conditions. On the other hand, health systems must be seen to preserve ease and equality of access to safe, timely and legal healthcare services in a non-discriminatory fashion. If liberal policies of toleration for CO are perceived to be detrimental in this respect, then the health system’s standing in the public’s eyes is likely to be damaged and trust may be eroded.

Policies and practical arrangements for CO can be said to be determined through a “negotiation” between key stakeholders: the health system in question, health professionals and their professional organizations, the public, and politicians. Notably, the latter group is likely to be very receptive to the views and concerns of the public. Similarly, for the reasons noted above, health systems must also be attentive to the views of the public in order to maintain their trust. In this context, it is important to understand the population’s attitudes to CO among health professionals.

### The Norwegian context

Norway has a liberal abortion law which provides for first trimester abortion on request. Abortions are performed in public hospitals, and the law gives health professionals a right to refuse to perform or assist in abortions. The question of whether general practitioners (GPs) should be allowed to refuse to *refer* for abortions became the focus of extensive debate in 2012–14 [6]. Judging from the public debate the public was predominantly opposed to CO to referrals. The political upshot of the debate was a new governmental policy in which GPs no longer have any right to CO in cases of abortion referral, or any other situation [7]. Assisted dying is illegal in Norway, yet in public debate it is often argued that if it were to be legalized, the law would have to provide for conscientious objectors. A previous analysis of the present study showed that 69% of the citizens polled supported CO for assisted dying, whereas 37% supported the present legal right to CO for abortion provision, and 32% would support a similar legal right to CO for abortion referrals [8].

### Potential determinants of attitudes towards assisted dying and conscientious objection

**Fig. 1:**
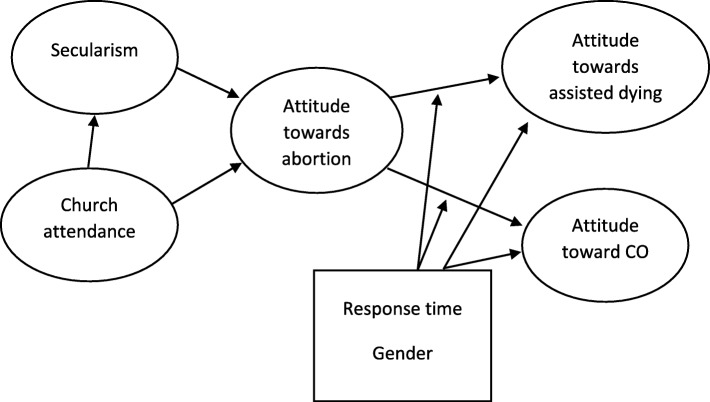
Determinants of attitudes towards assisted dying and objection. Conceptual model

In studies it is a robust finding that religiosity predicts opposition to abortion and assisted dying, and support for conscientious objection [9–11]. However, the *relationship* between the attitudes in play is typically not studied in depth. In this study, our hypothesis was that religiosity or secularism – defined as the attitude that society should build on non-religious values – are «fundamental attitudes» that are capable of predicting attitudes towards abortion, and that this trio of attitudes – religiosity, secularism, and abortion – would then predict attitudes to assisted dying and conscientious objection. For a typical citizen, then, attitudes to the two latter phenomena would to a large degree be determined by attitudes to abortion (see Fig. 1). The reasoning behind this prediction relates to the prominence of abortion as an ethical issue and the long-standing right to abortion on request. Abortion has been a recurring topic in public debates related to bioethics, often directly, yet also indirectly, as in, for instance, prenatal diagnosis. Thus, most people have established attitudes towards abortion. We therefore consider it likely that these attitudes will frame people’s thinking about newer medical ethics issues, such as assisted dying and conscientious objection.

## Methods

### Data and measures

The model was tested on data from NOBAS, the Norwegian Bioethics Attitude Survey, which assesses attitudes towards current bioethical issues in a representative sample of the population using a national web panel. In February 2017, a total of 1617 adult Norwegians completed the questionnaire (response rate: 8.5%; see Additional file 1 for the questionnaire). After removing missing data, the final sample included 1596 observations, which is well above the recommended sample size of 200 observations for the use of structural equation modelling (SEM) [12, 13]. Responses were weighted in order to mirror the demographic profile of the population. Table 1 reports the characteristics of the weighted sample (for unweighted sample, see Additional file 1: Appendix A).

**Table 1:** Demographic characteristics of respondents (*N* = 1596)

Characteristic		*N* (%)
Gender	Female	796 (49.8)
	Male	801 (50.2)
Age	18–24	190 (11.9)
	25–34	282 (17.7)
	35–44	283 (17.7)
	45–54	283 (17.7)
	> 54	558 (35.0)
Level of education	Primary school	74 (4.6)
	Upper secondary school	425 (26.6)
	College/university ≤3 years	434 (27.2)
	College/university > 3 years	643 (40.3)
	Unanswered	15 (0.9)
Religious beliefs	Non-religious	743 (46.5)
	Christian	678 (42.4)
	Other religions	26 (1.6)
	Unanswered	148 (9.3)

Attitude towards assisted dying, attitude towards conscientious objection, and attitude towards secularism were measured by two-item scales, while attitude towards abortion was measured by three items. All scale item questions used 5-point Likert scales anchored by 1 (fully disagree) and 5 (fully agree) (see Table 2 for all scale item questions and the relative reliability indices). Finally, we used church attendance as an indicator of religiosity and asked the respondents the following question: “How many times did you visit a church, house of prayer, mosque, synagogue etc. the last six months?” Church attendance is a common indicator of religiousness in social science research [15].

**Table 2:** Measures and Reliability of Latent Variables

Construct	Items	CR^a^	AVE^b^	α
Attitude towards Assisted Dying	• Assisted dying should be allowed for patients who are dying • Assisted dying should be allowed for patients who have an incurable chronic disease yet who are not dying	.82	.70	0.81
Attitude towards Conscientious Objection	• In general, healthcare professionals should be able to refrain from tasks for reasons of conscience, through local agreements that ensure the patient help from a colleague • In general, healthcare professionals should have a statutory right to refrain from tasks for reasons of conscience	.88	.78	0.87
Attitude towards Abortion	• In the first 12 weeks of pregnancy abortion should be available on request • The time limit for having an abortion should be extended to 16 weeks • It is ethically acceptable to choose abortion because the fetus has Down syndrome	.69	.43	0.67
Attitude towards Secularism	• Norwegian society should be secular, without influence from Christianity • Christianity should have greater influence on Norwegian society (reversed)	.82	.70	0.82

Notes: ^a^ *CR* Composite Reliability, ^b^ *AVE* Average Variance Extracted. They are computed based on formulas given by Fornell, Larcker [14]

### Statistical analyses

In order to test the interaction effects between different latent constructs in this study, we used the latent moderated structural equations (LMS) method developed by Klein and Moosbrugger [16]. The LMS approach is recommended as the tests based on the kurtosis and skewness statistics have shown that our data did not satisfy the multivariate normality assumption (e.g. [16, 17]). The approach utilizes the maximum likelihood robust estimation method in order to obtain standard errors that are more precise and robust than those provided by other approaches using product indicators. In addition, as the LMS approach does not require making any new indicator for the interaction term, it avoids potential confusion about the selection and formation of new product indicators (e.g. all-pairs vs. one-pair configurations) [18, 19].

Because the LMS model does not provide common model fit indices such as the comparative fit index (CFI), the Tucker-Lewis index (TLI), the root mean square error of approximation (RMSEA), and the Chi-square (*χ*^2^) value, we followed a two-step procedure (e.g. [16, 20]). Specifically, we first ran a structural equation model (SEM) without interaction effects (model 1). Secondly, we ran the full model including the hypothesized interaction effects (model 2). The likelihood ratio test was then used to examine whether the inclusion of the interaction effects improved the model fit. In both models, we applied the robust maximum likelihood estimation procedure using Mplus 7.2 [21].

Our results show that model 1 yields acceptable global goodness of fit indices: CFI = 0.930 and TLI = 0.900 are all above .9 [22], whereas RMSEA = 0.072 is below the recommended cut-off value of 0.08 [23, 24] and SRMR = 0.043 is well below 0.08 [13] (see Additional file 1: Appendix C for parameter estimates). Although the Chi-square statistic was significant (*χ*^2^(44) = 406.658, *p*-value = 0.000), it is acceptable as *χ*^2^ value is rather sensitive to minor departure from perfect fit when the sample is very large like in our case [22, 25]. As mentioned above, the full model with interaction (model 2) is estimated using the LMS approach so the relative fit of it versus model 1 was assessed through a likelihood ratio test comparing the log-likelihood values between the two models (see [20, 26]). Using a chi-square distribution, the likelihood ratio test proves significant (*χ*^2^(4)= 9.650, *p*-value < .05), indicating that the full model with the interaction terms is our optimal model in terms of fit. In other words, the inclusion of our studied interaction terms significantly improves our model’s ability to predict the outcome variables.

One could also argue that attitude towards secularism and church attendance could directly affect attitude towards assisted dying and conscientious objection. Therefore, we tested an alternative model (model 3) in which these direct effects were included. To account for all possibilities, we also allowed response time and gender to moderate the direct effects of attitude towards secularism and church attendance on attitudes towards assisted dying and conscientious objection. The chi-square difference test based log likelihood values show that model 3 significantly improves on model 2 in terms of fit (*χ*^2^(8)= 43.869, *p*-value < .001). Therefore, we chose model 3 to be our main model and describe the parameter estimates of this model in the next parts.

## Results

**Fig. 2:**
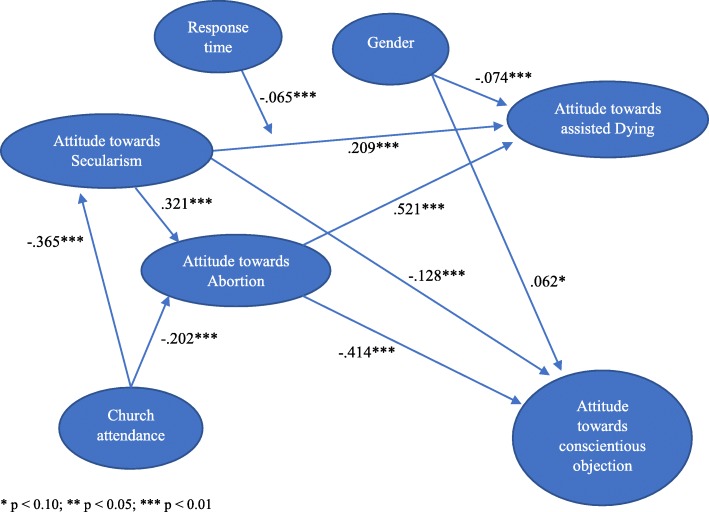
Main Structural Model (Model 3) with Standardized Coefficients shows the parameter estimates among our latent variables. *p* < 0.10; ** *p* < 0.05; *** *p* < 0.01

As shown in Fig. 2, attitude towards abortion is strongly and positively associated with attitude towards assisted dying. The path coefficient between these constructs is high and significant (β = 0.521, *p* < 0.01). Attitude towards abortion is negatively associated with attitude to CO. Again, the path coefficient is high and significant (β = − 0.414, *p* < 0.01). In turn, attitude towards abortion is positively associated with attitude towards secularism (β = 0.321, *p* < 0.01) and negatively associated with religiosity/church attendance (β = − 0.202, *p* < 0.01). We also observe strongly significant direct effects of attitude towards secularism on attitudes toward assisted dying and CO. In particular, attitude towards secularism is positively associated with attitude towards assisted dying (β = 0.209, *p* < 0.01) and negatively associated with attitude towards CO (β = − 0.128, *p* < 0.01). In addition, as expected, church attendance is negatively associated with attitude towards secularism (β = − 0.365, *p* < 0.01). Females are somewhat more negative towards assisted dying (β = − 0.074, *p* < 0.05) and more positive towards CO than men (β = 0.062, *p* = 0.05).

We observe a negative interaction effect of attitude towards secularism and response time on attitude towards assisted dying (β = − 0.065, *p* < 0.01), meaning that the (positive) relationship between attitude towards secularism and attitude towards assisted dying is somewhat weaker for respondents who spend more time on responding.

## Discussion

### Main findings summarized

The results show that pro-secular attitudes have a positive effect on attitudes towards assisted dying, and a negative effect on attitudes to CO. These effects are partially and largely mediated through established attitudes towards abortion. Religiosity (church attendance) has a positive effect on acceptance of CO and a negative effect on acceptance of assisted dying. These effects are fully mediated by established attitudes towards secularism and abortion.

### Conscientious objection is seen as tied to abortion

An important finding is that pro-secularity and support for abortion rights strongly influence attitudes towards both assisted dying and conscientious objection. In its essence, CO is a general phenomenon not necessarily tied to healthcare. Furthermore, it is only one of a set of dilemmas that arise for any pluralist liberal democracy concerning how the interests of moral and other minorities ought to be safeguarded. However, in the Norwegian consciousness CO appears to have become closely associated with abortion. This might be a result of recent Norwegian public debate on CO, which has been scant except in 2012–2014 when the topic of CO to general practitioners’ referrals for abortion figured very prominently on the political agenda, as mentioned above. A large majority of Norwegians favour liberal abortion rights including abortion on request in the first trimester.

### Conscientious objection, secularity and tolerance

As attitudes to abortion are apparently a strong determinant of attitudes to conscientious objection, then from the perspective of a defender of conscientious objection it was quite unfortunate that the topic came to be so closely tied to abortion. Our findings support the interpretation that conscientious objection conceivably could have received greater public support if abortion had not become the “test case” in the public debate. Supporters of CO would do well to attempt to untie the debate from the specific topic of abortion, showing CO to be a more general and principled question not antithetical to abortion rights. Following the same logic, opponents of assisted dying could have an interest in disentangling the topic from abortion and from religion, whereas proponents of assisted dying could benefit from rendering assisted dying as, for instance, belonging to the same struggle for increased personal freedom as did the struggle for abortion rights.

Building on our findings a further hypothesis could be that the more secular the population becomes, the lower the *tolerance for CO* becomes. If religiosity wanes and pro-secular and pro-abortion attitudes become more widespread, support for conscientious objection might decline, putting into question whether any present policies of toleration of conscientious refusals as a practical compromise will remain acceptable to the majority. Potentially, health systems that continue to uphold a broad toleration for conscientious refusals might then endanger their standing with, and trust from, the public.

Arguably, the precise *way* tolerance for objectors is implemented in practice is decisive for whether it actually conflicts with patient rights and interests concerning access to care. In principle, conscientious objection might place four significant kinds of burdens on patients: there could be delays or extra expenses in receiving care; access to care could be restricted; patients could fail to receive relevant information about care choices; and the encounter with the objector or the way the objection is communicated could lead to the patient perceiving moral disapproval of their lifestyle or healthcare choices [27]. Thus, for policies of conscientious objection to be acceptable to healthcare systems and to patients, care must be taken to avoid or minimize burdens in all four domains.

Apparently, respondents who favour secularism became less positive towards assisted dying when they spent more time on responding. Although speculative, one interpretation could be that secularism for many involves a strong emphasis on individual freedom, making it likely that one’s first thought about the issue of assisted dying is a strong endorsement. Counter-arguments in the complex issue of assisted dying, however, are not as strongly tied to secularism and might require more time for reflection to take fully into account.

### Limitations

It is not possible to draw firm conclusions on the nature of causal relationships based solely on analyses of cross-sectional survey data. The best method for testing causal relationships is randomized experimentation. However, the variables involved in this study are difficult to manipulate in controlled experiments. This is why we use the second-best alternative: a version of structural equation modeling [28, 29]. With this approach, we test how alternative models, which all make sense from a theoretical perspective, fit with the covariance structure in the dataset. We need to argue theoretically for the direction of relationships. The revised model fits very well with the covariance structure and the theoretical arguments for the relationships are reasonable. Still, there is uncertainty involved in the causal reasoning. In particular, there could be other mediators and/or moderators.

The study examines the attitudes of Norwegians and it is therefore an open question whether the same tendencies are operative in other Western countries. It would be valuable to conduct similar studies in other countries.

The low response rate means that a non-response bias cannot be excluded. However, data have been weighed in order to more closely mirror population demographics.

## Conclusion

This study on the relationship between attitudes among the Norwegian public has indicated that support for abortion rights is associated with pro-secular attitudes and is a main “driver” for support for assisted dying and opposition to conscientious objection. This finding should be regarded as a hypothesis which ought to be tested in other populations. If the relationship is robust and reproduced elsewhere, there are important consequences for CO advocates who would then have an interest in disentangling the debate about CO from abortion; and for health systems who ought to consider carefully how a sound policy on CO can safeguard both patient trust in the services and the moral integrity of professionals.

## Supplementary information

**Additional file 1:** **Appendix A.** Demographic characteristics of respondents; **Appendix B.** Confirmatory Factor Analysis; **Appendix C.** Model 1 (without interaction terms) with Standardized Coefficients; **Appendix D.** The NOBAS questionnaire, translated into English.

## Data Availability

The data are available on reasonable request to the first author.

## Abbreviations

CFI: Comparative fit index
CO: Conscientious objection
LMS: Latent moderated structural equations
NOBAS: Norwegian Bioethics Attitude Survey
RMSEA: Root mean square error of approximation
SEM: Structural equation modelling
SRMR: Standardized root mean residual
TLI: Tucker-Lewis index
