# Supplementary material for: Secularity, abortion, assisted dying and the future of conscientious objection: modelling the relationship between attitudes
Source: BMC Med Ethics. 2019 Sep 18;20:65. doi: 10.1186/s12910-019-0408-4 (PMC6751575; doi:10.1186/s12910-019-0408-4)
Supplement: Supplementary file 1 — Additional file 1: Appendix A. Demographic characteristics of respondents; Appendix B. Confirmatory Factor Analysis; Appendix C. Model 1 (without interaction terms) with Standardized Coefficients; Appendix D. The NOBAS questionnaire, translated into English. [file 12910_2019_408_MOESM1_ESM.docx]

**Online appendices**

**Appendix A:** Demographic characteristics of respondents (N = 1596)

| Characteristic | | Unweighted (N, (%)) | Weighted (N, (%)) |
| --- | --- | --- | --- |
| Gender | Female | 802 (50.3) | 796 (49.8) |
|  | Male | 794 (49.7) | 801 (50.2) |
| Age | 18-24 | 109 (6.8) | 190 (11.9) |
|  | 25-34 | 233 (14.6) | 282 (17.7) |
|  | 35-44 | 248 (15.5) | 283 (17.7) |
|  | 45-54 | 305 (19.1) | 283 (17.7) |
|  | > 54 | 701 (43.9) | 558 (35.0) |
| Level of education | Primary school | 74 (4.6) | 74 (4.6) |
|  | Upper secondary school | 391 (24.5) | 425 (26.6) |
|  | College/university ≤ 3 years | 451 (28.3) | 434 (27.2) |
|  | College/university > 3 years | 654 (41.0) | 643 (40.3) |
|  | Unanswered | 18 (1.1) | 15 (0.9) |
| Religious beliefs | Non-religious | 719 (45.1) | 743 (46.5) |
|  | Christian | 702 (44.0) | 678 (42.4) |
|  | Other religions | 26 (1.6) | 26 (1.6) |
|  | Unanswered | 147 (9.2) | 148 (9.3) |

**Appendix B:** Confirmatory Factor Analysis

**Table B1: Measures and Factor Loadings**

| **Construct** | **Items** | **Standardized Factor loadings** |
| --- | --- | --- |
| Attitude towards Assisted Dying | • Assisted dying should be allowed for patients who are dying | 0.913 |
|  | • Assisted dying should be allowed for patients who have an incurable chronic disease yet who are not dying | 0.748 |
| Attitude towards Conscientious Objection |  | 0.83 |
|  | - In general, healthcare professionals should be able to refrain from tasks for reasons of conscience, through local agreements that ensure the patient help from a colleague   • In general, healthcare professionals should have a statutory right to refrain from tasks for reasons of conscience | 0.936 |
| Attitude towards Abortion | • In the first 12 weeks of pregnancy abortion should be available on request | 0.58 |
|  | • The time limit for having an abortion should be extended to 16 weeks | 0.749 |
|  | • It is ethically acceptable to choose abortion because the fetus has Down syndrome | 0.624 |
| Attitude towards Secularism | • Norwegian society should be secular, without influence from Christianity | 0.802 |
|  | • Christianity should have greater influence on Norwegian society (reversed) | 0.866 |
| **Notes:** All factor loadings are significant at p < .001  Model fits: Satorra-Bentler Scaled$\chi^{2}$ (df = 31) = 243.596 (p-value < .001), RMSEA = 0.066, CFI = 0.958, TLI = 0.925, SRMR = 0.036 | | |

**Table B2: Correlation matrix of latent constructs**

|  | SECU | ABO | AD | CO |
| --- | --- | --- | --- | --- |
| SECU | 1.000 |  |  |  |
| ABO | 0.387 (0.036) | 1.000 |  |  |
| AD | 0.392 (0.029) | 0.588 (0.031) | 1.000 |  |
| CO | -0.301 (0.029) | -0.435 (0.036) | -0.326 (0.029) | 1.000 |
| **Notes:** Values within parentheses are standard errors.  SECU: attitude towards secularism, ABO: attitude towards abortion, AD: attitude towards assisted dying, CO: attitude towards conscientious objection | | | | |

**Appendix C:** Model 1 (without interaction terms) with Standardized Coefficients

-.365***

-.209***

.397***

.638***

-.495***

-.064**

.039*

n.s.

.051**

* p < 0.10; ** p < 0.05; *** p < 0.01

$\chi^{2}\left( 44 \right)$ = 406.658 (p < .001); CFI = 0.930; TLI = 0.900; SRMR = 0.043; RMSEA = 0.072

**Appendix D:** The NOBAS questionnaire, translated into English

**Section 1: Conscientious objection**

Introduction version 1: ‘Freedom of conscience is the right to act according to your own conscience. Freedom of conscience is considered a fundamental human right. To what extent should society display tolerance? Should workers have the right to refrain from certain tasks at work due to conscience?’

Introduction version 2: ‘Occasionally, workers refuse to perform certain duties due to their conscience. Where should the society set the limit? Should workers have an unconditional right to conscientious objection to certain tasks?’

Respondents were randomized to receive either version 1 or 2.

First set of questions:

Q1.  In general, healthcare professionals should be able to refrain from tasks for reasons of conscience, through local agreements that ensure the patient help from a colleague.

Q2.  In general, healthcare professionals should have a statutory right to refrain from tasks for reasons of conscience.

Q3. Doctors should be able to refrain from performing ritual circumcision of infant boys.

Q4. If assisted dying is legalized in Norway, doctors should be able to refrain from performing this.

Second set of questions:
Introduction: A [gender] doctor wants to refrain from [procedure]. [Religious affiliation]
(*Variables:* Gender: female/male. Procedure: performing abortion/referring for abortion. Religious affiliation: The doctor is Muslim/The doctor is Christian/(no information given))

Q5.  The doctor’s wish to refrain should be respected, as long as it can be facilitated in practice and the patient is ensured help from a colleague.

Q6.  Doctors should have a statutory right to conscientious objection in such cases.

Response alternatives for all questions: Fully disagree, somewhat disagree, neither agree nor disagree, somewhat agree, fully agree.

**Section 2: Prenatal diagnosis**

Introduction: Prenatal diagnosis by ultrasound and blood tests in pregnancy weeks 11-13 is used in particular to detect Down syndrome or other chromosomal abnormalities in the fetus. This examination is currently offered to pregnant women with the highest risk, which means especially those over 38 years of age. Some believe that the examination should be offered to all pregnant women.

Q1. Prenatal diagnosis by ultrasound and blood test in pregnancy week 11-13 should be offered to all pregnant women, without co-payment

Q2. I think pregnant women whose fetus has been shown to have Down syndrome often experience pressure and expectations that they choose abortion

Q3. It is ethically acceptable to choose abortion because the fetus has Down syndrome

Q4. An offer of prenatal diagnosis to all pregnant women will send a signal that people with Down syndrome are unwanted in society

Q5. It is ethically more problematic to choose abortion when the child was initially planned and desired (as it usually is in prenatal testing) than when the pregnancy was unplanned and unwanted

Q6. A society that offer prenatal diagnosis to all pregnant women, can be criticized for being a sorting society

Q7. Those whose fetus has been diagnosed with Down syndrome, but still complete the pregnancy, should not expect extensive assistance from the state

Q8. When the fetus has Down syndrome, the pregnant woman should have an ethical obligation to choose abortion

Q9. Abortion is acceptable if the fetus has a disorder that would place a heavy burden of care on the family

Q10. I think pregnant women whose fetus has been shown to have Down syndrome often experience pressures and expectations that they complete pregnancy (that is, *not* to choose abortion)

Q11. In the future it might be possible to map all fetal genes through a blood test of the mother in early pregnancy. Consider the claim: If such a blood test becomes available, it should be legal in Norway for all pregnant women

(Response alternatives for all questions: Fully disagree, somewhat disagree, neither agree nor disagree, somewhat agree, fully agree.)

**Section 3: Religious framing**

(Response alternatives for all questions in this section: Fully disagree – somewhat disagree – neither agree nor disagree – somewhat agree – fully agree)

**Introductory text**

You will now receive questions about assisted dying, use of surrogate motherhood, and abortion on request, and about some of the arguments used in the debates.

**Assisted dying** (respondents were randomized to receive one of three versions)

Version 1:

Assisted dying means a doctor taking a person’s life by injection lethal medication after the person’s request, or procures lethal medication that the person can ingest themselves. Assisted dying is illegal in Norway.

Christian organizations arguing against assisted dying claim that legalization can lead to weak groups experiencing pressure to request assisted dying.

Version 2:

Assisted dying means a doctor taking a person’s life by injection lethal medication after the person’s request, or procures lethal medication that the person can ingest themselves. Assisted dying is illegal in Norway.

Some who argue against assisted dying claim that legalization can lead to weak groups experiencing pressure to request assisted dying.

Version 3:

Assisted dying means a doctor taking a person’s life by injection lethal medication after the person’s request, or procures lethal medication that the person can ingest themselves. Assisted dying is illegal in Norway.

Christian organizations arguing against assisted dying claim that legalization can lead to weak groups experiencing pressure to request assisted dying. This would go against the Christian duty to protect the weak.

**Questionnaire** (the same for the three versions of the introduction):

Consider the claims:

1. Assisted dying should be allowed for patients who are dying
2. Assisted dying should be allowed for patients who have an incurable chronic disease yet who are not dying
3. Legalization of assisted dying can lead to weak groups experiencing pressure to request assisted dying.

**Use of surrogate motherhood** (not shown here)

**Abortion on request**

Version 1:

In Norway the woman herself decides whether to have an abortion, up to and including the 12th week of pregnancy (abortion on request). Sometimes there are proposals to amend the time limit for abortion. The Socialist Left Party (SV) want abortion on request up to and including week 16.

Version 2:

In Norway the woman herself decides whether to have an abortion, up to and including the 12th week of pregnancy (abortion on request). Sometimes there are proposals to amend the time limit for abortion. The Christian Democrat Party (KrF) want to restrict the right to abortion on request.

Consider the claims:

1. In the first 12 weeks of pregnancy abortion should be available on request
2. The time limit for having an abortion should be extended to 16 weeks

**Section 4: Patients’ and next of kin’s participation in decision-making in medicine** (not shown here)

**Section 5: Questionnaire psychology and demographic background variables**

1. What is your highest completed education? (Alternatives: Primary school, upper secondary school, College/university ≤ 3 years, College/university > 3 years, unanswered)
2. Which of the alternatives below best descripes your worldview? (a. Christian; b. Muslim; c. Other religious worldview; d. Atheist/agnostic; e. Another non-religious worldview; f. Prefer not to state)
3. How many times in the last six months have you been to church, house of worship, mosque, synagogue, etc? (a. Never; b. 1-6 times last 6 months.; c. 1-3 times per month.; d. >3 times per month; e. Prefer not to state)
4. Christianity should have greater influence on Norwegian society (Fully disagree – somewhat disagree – neither agree nor disagree – somewhat agree – fully agree)
5. Norwegian society should be secular, without influence from Christianity (Fully disagree – somewhat disagree – neither agree nor disagree – somewhat agree – fully agree)
6. How easy or difficult did you find it to answer the ethial questions in this survey?

(1=very easy, 7=very difficult).

This survey encompasses several areas within medical ethics. Some of the areas can be perceived as more important than others. How important are the different areas in your opinion?

1. Abortion (1=not important, 7=very important)
2. Assisted dying
3. Conscientious objection for doctors
4. Prenatal diagnosis
5. Patients’ and next of kin’s participation in decision-making in medicine
